# Sindbis and Middelburg Old World Alphaviruses Associated with Neurologic Disease in Horses, South Africa

**DOI:** 10.3201/eid2112.150132

**Published:** 2015-12

**Authors:** Stephanie van Niekerk, Stacey Human, June Williams, Erna van Wilpe, Marthi Pretorius, Robert Swanepoel, Marietjie Venter

**Affiliations:** University of Pretoria, Pretoria, South Africa (S. van Niekerk, S. Human, J. Williams, E. van Wilpe, M. Pretorius, R. Swanepoel, M. Venter);; US Centers for Disease Control and Prevention, Pretoria, South Africa (M. Venter).

**Keywords:** Alphaviruses, Sindbis virus, Middelburg virus, horses, neurologic disease, RT-PCR, South Africa, viruses

## Abstract

Old World alphaviruses were identified in 52 of 623 horses with febrile or neurologic disease in South Africa. Five of 8 Sindbis virus infections were mild; 2 of 3 fatal cases involved co-infections. Of 44 Middelburg virus infections, 28 caused neurologic disease; 12 were fatal. Middelburg virus likely has zoonotic potential.

Alphaviruses (*Togaviridae*) include zoonotic, vectorborne viruses with epidemic potential (*1*). Phylogenetic analysis defined 2 monophyletic groups: 1) the New World group, consisting of Sindbis virus (SINV), Venezuelan equine encephalitis virus, and Eastern equine encephalitis virus; and 2) the Old World group, consisting of Semliki Forest virus (SFV), Middelburg virus (MIDV), Ndumu virus, Chikungunya virus (CHIKV), and Barmah Forest virus (*2*). Old World alphaviruses are associated mainly with febrile disease and arthralgia, are often accompanied by a maculopapular rash, and are rarely fatal, although neurologic cases have been reported (*3*). In contrast, New World alphaviruses are associated with neurologic disease in horses and, potentially, humans (*4*).

We previously investigated horses as sentinels for detection of neurologic arboviruses and described West Nile virus (WNV) lineage 2 (*5*) and Shunivirus (SHUV) as previously missed causes of fatal encephalitis in Africa (*6*), with zoonotic potential (*7*). Five alphaviruses have been detected in vectors in southern Africa: SINV, CHIKV, MIDV, Ndumu virus, and SFV (*8*); however, little is known about prevalence, pathogenicity, and host range (*9*).

## The Study

To determine if alphaviruses may contribute to undefined neurologic infections, we investigated specimens (blood, cerebrospinal fluid, or tissue from brain, spinal cord, or visceral organs) from 623 horses with unexplained febrile and acute neurologic infections reported to our surveillance program by veterinarians across South Africa during January 2008–December 2013. Of reported cases, 346 horses had neurologic signs; 277 had mainly febrile illness and other miscellaneous signs, including colic and sudden death (Technical Appendix Figure 1). Formalin-fixed tissue samples from horses that died were submitted for histopathology. Horses ranged from <1 to 20 years of age and included thoroughbred, Arabian, warm-blood, and part-bred horses; most were bred locally.

A generic nested alphavirus nonstructural polyprotein (nsP) region 4 gene reverse transcription PCR (*10*) was used to screen total nucleic acids. TaqMan probes (Roche, Indianapolis, IN, USA) were developed for rapid differentiation of MIDV and SINV by real-time PCR (online Technical Appendix).

**Figure 1:**
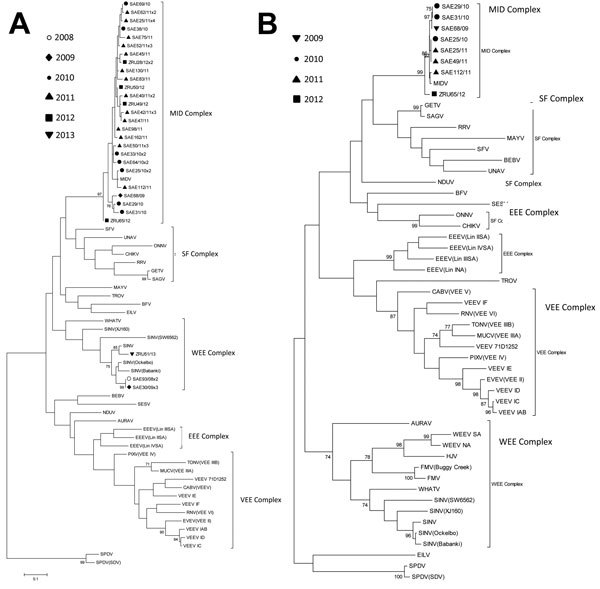
Maximum-likelihood trees of strains of Middelburg virus and Sindbis virus identified in horses in South Africa relative to other members of the alphavirus genus. Trees were constructed by using the Tamura-Nei substitution model and midpoint rooted with MEGA5 (http://www.megasoftware.net/). Scale bar indicates 0.1 nt substitutions. Estimates were constructed on the basis of bootstrap resampling performed with 1,000 replicates. Confidence estimates >70 are shown. A) A 200-bp fragment of the nonstructural polyprotein region 4 gene of MDV- and SINV-positive cases. B) A 348-bp fragment of the E1 gene of 7 MIDV cases identified in horses in southern Africa (genome position 10543–10911 corresponding with the MIDV-857 strain in GenBank accession no. EF536323). Reference sequences used in these trees are as previously described (*2*). Complexes are identified as follows: EEE, Eastern equine encephalitis; MID, Middelburg; SF, Semliki Forest; VEE, Venezuelan equine encephalitis; WEE, Western equine encephalitis. Viruses are identified as follows: AURAV, Aura virus; BEBV, Bebaru virus; BFV, Barmah Forest virus; CABV, Cabassou virus; CHIKV, Chikungunya virus; EEEV, Eastern equine encephalitis virus; EILV, Eilat virus; EVEV, Everglades virus; FMV, Fort Morgan virus; GETV, Getah virus; HJV, Highlands J virus; MAYV, Mayaro virus; MIDV, Middelburg virus; MUCV, Mucambo virus; NDUV, Ndumu virus; ONNV, O’nyong nyong virus; PIXV, Pixuna virus; RNV, Rio Negro virus; RRV, Ross River virus; SAE, South Africa equine virus; SAGV, Sagiyama virus; SESV, Southern elephant seal virus; SFV, Semliki Forest virus; SINV, Sindbis virus; SPDV, Salmon pancreatic disease virus; TONV, Tonate virus; TROV, Trocara virus; UNAV, Una virus; VEEV, Venezuelan equine encephalitis virus; WEEV, Western equine encephalitis virus; WHATV, Whataroanvirus; ZRU, Zoonoses Research Unit virus.

PCR-positive cases were confirmed by sequencing the nsP4 amplicon (200 bp), followed by maximum-likelihood analysis (Figure 1, panel A). Additional amplification, sequencing, and phylogenetic analysis of a 349-bp E1 gene fragment (online Technical Appendix) was attempted (Figure 1, panel B) to investigate recombination events.

**Figure 2:**
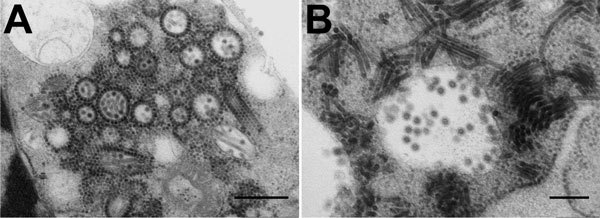
Electron micrographs of Middelburg virus isolate SAE25/2011 in baby hamster kidney cell culture. A) Several enveloped virions consisting of a dense core and surrounded by a translucent layer are shown in the vesiculated endoplasmic reticulum. The virus has elongated forms and numerous precursor nucleocapsids in the cytoplasm. Many of the nucleocapsids are associated with the outer surfaces of the vesiculated endoplasmic reticulum. Scale bar indicates 500 nm. B) Virions in a cytopathic vacuole are surrounded by elongated forms of the virus. Scale bar indicates 200 nm. Micrographs courtesy of Stephanie van Niekerk et al.

Baby hamster kidney cell–culture isolates were obtained for 2 MIDV strains under Biosafety Level 3 conditions. Isolate SAE25/2011 from blood of a horse with neurologic signs was visualized by electron microscopy (Figure 2); the full genome was sequenced as described (*11*). By using maximum-likelihood and P-distance analysis (Technical Appendix Figure 2), we compared isolate SAE25/2011 with MIDV-857, which was isolated in 1993 from spleen of a horse with African horse sickness virus (AHSV)–like signs in Zimbabwe (GenBank accession no. EF536323) (*12*). Differential diagnosis for flaviviruses, WNV, Wesselsbron virus, SHUV, and equine encephalosis viruses was performed on all specimens, which were sent to other laboratories for testing for AHSV, equine herpes viruses, and rabies (*5*,*6*).

Of the 623 horses, 8 (1.3%) tested positive for SINV and 44 (7.1%) for MIDV (Table 1). Of the 8 horses with SINV, 3 survived febrile illness, 2 survived neurologic disease, and 3 died from neurologic disease. Two of the 3 horses that died with SINV had WNV co-infection and were positive by PCR for both viruses in brain tissue. In those 2 horses, lesions of meningoencephalitis were visible by histopathology but were nonspecific and could not be ascribed to either virus. Immunohistochemistry (IHC) for WNV in equine cases is reportedly a poor diagnostic tool (*13*), although IHC for SINV and MIDV needs to be established. In the third fatal case, SINV was detected in blood; neurologic samples were unavailable for testing. One horse with SINV had mild colic, dysphagia with tongue paralysis, and pale mucus membranes (Table 2).

**Table 1:** Prevalence of neurologic and febrile cases of MIDV and SINV infections in horses, South Africa, 2008–2013*

Alphavirus results	2008	2009	2010	2011	2012	2013	Total
MIDV							
Specimens, no.	76	50	137	166	107	100	636 (100)
PCR+, no. (% of total specimens)	0	1 (2)	10 (7)	26 (16)	4 (4)	3 (3)	44 (7)
Deaths, no. (% of no. PCR+)	NA	0	2 (50)	7 (27)	3 (75)	0	12 (27)
Detected in CNS, no. (% of no. PCR+)	NA	0	0	6 (23)	0	0	6 (14)
Neurologic disease, no. (% of no. PCR+)	NA	1	7 (70)	16 (62)	4 (100)	2 (67)	30 (68)
Fever only, no. (% of no. PCR+)	NA	0	6 (60)	5 (19)	2 (50)	0	13 (30)
Co-infections, no. and type	NA	0	1 SHUV, 1 EEV	1 WNV,† 1 EEV, 1 AHSV†	0	0	2 EEV, 1 ASHV,† 1 SHUV, 1 WNV†
SINV							
Specimens, no.	76	50	137	166	107	100	636
SINV+, no. (% of total specimens)	2 (3)	4 (8)	0	0	1 (1)	1 (1)	8 (1)
Deaths, no. (% of no.+)	0 (0)	2† (50)	NA	NA	1 (100)	0	3 (38)
Detected in CNS, no. (% of no.+)	–	2 (50)	NA	NA	0	NA	
Neurologic disease, no. (% of no.+)	1 (50)	2 (50)	NA	NA	1 (100)	0	4 (50)
Fever only, no. (% of no.+)	1 (50)	2 (50)	NA	NA	0	1 (100)	4 (50)
Co-infections, no. and type	1 EEV, 1 AHSV	2 WNV†	NA	NA	0	0	1 EEV, 1 AHSV, 2 WNV†

*AHSV, African horse sickness virus; CNS, central nervous system sample; EEV, equine encephalosis virus; MIDV, Middelburg virus; NA, not applicable; SHUV, Shunivirus; SINV, Sindbis virus; WNV, West Nile virus; –, undetectable in central nervous system sample; +, positive. †Fatal cases with co-infections.

**Table 2:** Clinical signs associated with MIDV and SINV infections in horses, South Africa, 2008–2013*

Clinical sign	SINV, no. (%), n = 8	MIDV, no. (%), n = 44
Fever	5 (62.5)	20 (45.5)
Ataxia	2 (25.0)	16 (36.4)
Unspecified neurologic signs	2 (25.0)	14 (31.8)
Muscle fasciculation	0	4 (9.0)
Muscle weakness	1 (12.5)	2 (5.0)
Depression, listlessness	1 (12.5)	10 (22.7)
Hepatitis/Icterus	0	1 (2.0)
Limb paresis, paralysis	0	7 (15.9)
Recumbency	2 (25.0)	6 (13.6)
Tachycardia	0	6 (13.6)
Tachypnea, dyspnea	0	5 (11.4)
Fasciculations	0	4 (9.1)
Seizures	0	2 (4.5)
Abortion	0	1 (2.0)
*MIDV, Middelburg virus; SINV, Sindbis virus.		

Of 44 horses with MIDV, 16 had febrile disease, 28 had neurologic disease, and 12 died. Five dual infections were detected: 1 horse with MIDV and SHUV infections and 2 with MIDV and EEV infections survived; 1 horse with MIDV and AHSV infections and 1 with MIDV and WNV infections died (Table 2). For 6 of 12 horses that died with MIDV, central nervous system samples were available, and MIDV was detected in brain of all 6 horses. AHSV was detected in lung and spleen and MIDV in brain of 1 horse. MIDV and WNV was found in brain of another; only MIDV was detected in brain of the other 4.

Altogether, 26 (66.7%) of 39 horses with MIDV single infection showed signs of neurologic disease (Table 2). Clinical signs with less severe illness included fever, stiffness, swollen limbs, hyperreactiveness, and depression; signs of severe neurologic disease included ataxia, paresis, paralysis, recumbency, and seizures. One horse exhibited icterus, 1 had a pregnancy abort, and 1 had laminitic stance. Brain and cord tissues from 4 horses with neurologic disease caused by MIDV single infections were examined microscopically. MIDV was detected in blood of 1 horse but in brain of the others. Lesions of mild to moderate meningoencephalitis were observed in all 4, including perivascular cuffing involving mainly mononuclear cells, glial nodules, and diffuse gliosis. 

No clear associations with age, gender, or breed were apparent for either virus. Number of cases peaked during the rainy season (February–May), consistent with vectorborne diseases, although sporadic cases were detected year-round (Technical Appendix Figure 1, panel B). Both viruses were widely distributed across South Africa (Technical Appendix Figure 3).

The nsP4 gene fragment for 6 SINV-positive specimens had <7% nt differences but 100% aa identity, clustering closely to the Ockelbo strain from Sweden and Babanki strain from Cameroon. One SINV isolate was of unrecorded origin (Figure 1, panel A) (*2*).

MIDV nsP4 partial sequences of 26 unique strains clustered with MIDV-857 (*12*), (Figure 1, panel A), with 3.8% nt and 2.8% aa differences from South Africa strains and <5% nt and 3.6% aa differences from MIDV-857. No specific relationships to time, geographic origin, or outcome were evident, although the length and conserved nature of the diagnostic amplicon limited the phylogenetic analysis. The E1 gene fragments from 7 MIDV cases (GenBank accession nos. JN226792–JN226795, KF680222–KF680224) clustered with MIDV-857 (Figure 1, panel B), differing by ≤1.2% nt and 0.5% aa levels from each other and by ≤1.4% nt and 0.6% aa levels from MIDV-857.

The genome of MIDV isolate SAE25/2011 (GenBank accession no. KF680222) was 11,674 nt in length, excluding the poly (A) tail, with 98.5% nt and 99.4% aa identity to MIDV-857 (*12*), clustering similarly as previous alphavirus phylogenetic investigations (*2*). Seven aa differences exist in the structural polyprotein (98.7% identity) and 17 in the nsP region (99.3% identity). Three aa changes in the nsP and 3 in the structural polyprotein altered hydrophilicity. The nsP1 amino acid sequences differed by 0.8%; the nsP2 and nsP3 were identical; and nsP4 differed by 0.5%. The capsid protein differed by 1.1%; E1 and E2 differed by 0.5%; and E3 and 6K proteins were identical. SAE25/2011, like MIDV-857, also contained the recombinant SFV domains identified previously (*12*).

## Conclusions

Besides CHIKV, SINV is the most widely distributed Old World alphavirus. It is associated with fever, rash, and arthralgia in humans in Europe, Asia, Africa, and Australia (*8*) and has been isolated from mosquitoes, birds, and humans in South Africa. Although antibodies described in livestock and horses, it has been unrecognized as a potential pathogen of horses. Less is known about MIDV. Isolated in 1957 from mosquitoes in South Africa, subsequent surveys identified antibodies in humans, horses, and livestock, and the virus has been isolated in mosquitoes and 2 humans elsewhere in Africa (*14*). In 1974, MIDV was isolated from blood of a horse during an outbreak of fever and icterus and was implicated in a horse with anorexia and muscular stiffness in South Africa (*14*) and from spleen of a horse in Zimbabwe in 1993 (*11*). Additional investigations have been limited, and the virus has not previously been associated with neurologic disease.

This study was not structured to provide definitive information on the prevalence and incidence of disease and likely underestimates the situation. Development of serologic assays for diagnosis of cases past the viremic stage are needed to establish the true prevalence and effects of disease, and IHC is needed for investigating the pathogenesis in horses and other species. Our findings strongly suggest that Old World alphaviruses, particularly MIDV, may constitute an overlooked cause of febrile and neurologic disease in horses and, like New World alphaviruses, may pose threats to horses, livestock, and humans.

**Technical Appendix.** Detailed real-time PCR methods used for testing samples for Middelburg virus and Sindbis virus infections identified in horses in South Africa and figures showing seasonal and geographic distribution.

## References

[1] Calisher CH, Karabatsos N. Arbovirus serogroups: definition and geographic distribution. In: Monath TP, editor. The arboviruses: epidemiology and ecology. Baca Raton (FL): CRC Press; 1988. p. 19–57.

[2] Forrester NL, Palacios G, Tesh RB, Savji N, Guzman H, Sherman M, Genome-scale phylogeny of the alphavirus genus suggests a marine origin. J Virol. 2012;86:2729–38. 10.1128/JVI.05591-11 22190718 PMC3302268

[3] Lewthwaite P, Vasanthapuram R, Osborne JC, Begum A, Plank JL, Shankar MV, Chikungunya virus and central nervous system infections in children, India. Emerg Infect Dis. 2009;15:329–31. 10.3201/eid1502.080902 19193287 PMC2662654

[4] Weaver SC, Winegar R, Manger ID, Forrester NL. Alphaviruses: population genetics and determinants of emergence. Antiviral Res. 2012;94:242–57. 10.1016/j.antiviral.2012.04.002 22522323 PMC3737490

[5] Venter M, Human S, Zaayman D, Gerdes GH, Williams J, Steyl J, Lineage 2 West Nile virus as cause of fatal neurologic disease in horses, South Africa. Emerg Infect Dis. 2009;15:877–84. 10.3201/eid1506.081515 19523285 PMC2727306

[6] van Eeden C, Williams JH, Gerdes TG, van Wilpe E, Viljoen A, Swanepoel R, Shuni virus as cause of neurologic disease in horses. Emerg Infect Dis. 2012;18:318–21. 10.3201/eid1802.111403 22305525 PMC3310469

[7] van Eeden C, Swanepoel R, Venter M. Antibodies against West Nile and Shuni Viruses in Veterinarians, South Africa. Emerg Infect Dis. 2014;20:1409–11. 10.3201/eid2008.131724 25062350 PMC4111201

[8] Lloyd G. Alphaviruses. In: Zuckerman AJ, Banatvala JE, Griffiths PD, Schoub B, Mortimer P, editors. Principles and practices of clinical virology. West-Sussex (UK): Wiley-Blackwell; 2009. p. 643–68.

[9] Storm N, Weyer J, Markotter W, Kemp A, Leman PA, Dermaux-Msimang V, Human cases of Sindbis fever in South Africa, 2006–2010. Epidemiol Infect. 2014;142:234–8. 10.1017/S0950268813000964 23611492 PMC9151170

[10] Sánchez-Seco MP, Rosario D, Quiroz E, Guzmán G, Tenorio A. A generic nested-RT-PCR followed by sequencing for detection and identification of members of the alphavirus genus. J Virol Methods. 2001;95:153–61. 10.1016/S0166-0934(01)00306-8 11377722

[11] van Eeden C, Harders F, Kortekaas J, Bossers A, Venter M. Genomic and phylogenetic characterization of Shuni virus. Arch Virol. 2014;159:2883–92. 10.1007/s00705-014-2131-2 24957652

[12] Attoui H, Sailleau C, Mohd Jaafar F, Belhouchet M, Biagini P, Cantaloube JF, Complete nucleotide sequence of Middelburg virus, isolated from the spleen of a horse with severe clinical disease in Zimbabwe. J Gen Virol. 2007;88:3078–88. 10.1099/vir.0.83076-0 17947533

[13] Williams JH, van Niekerk S, Human S, van Wilpe E, Venter M. Pathology of fatal lineage 1 and 2 West Nile virus infections in horses in South Africa. J S Afr Vet Assoc. 2014;85:1105. 10.4102/jsava.v85i1.1105 25686260

[14] McIntosh BM. The epidemiology of arthropod-borne viruses in Southern Africa [dissertation]. Pretoria (SA): University of Pretoria; 1980.

