# Supplementary material for: Sindbis and Middelburg Old World Alphaviruses Associated with Neurologic Disease in Horses, South Africa
Source: Emerg Infect Dis. 2015 Dec;21(12):2225–9. doi: 10.3201/eid2112.150132 (PMC4672445; doi:10.3201/eid2112.150132)
Supplement: Supplementary file 1 — Technical Appendix. Detailed real-time PCR methods used for testing samples for Middelburg virus and Sindbis virus infections identified in horses in South Africa and figures showing seasonal and geographic distribution. [file 15-0132-Techapp-s1.pdf]

Article DOI: http://dx.doi.org/10.3201/eid2112.150132

Sindbis and Middelburg Old World Alphaviruses Associated with Neurologic Disease in Horses, South Africa

Technical Appendix

Detailed Methods and Results

1. Generic Real-Time Reverse Transcription PCR for Diagnosis of Middelburg and Sindbis Virus Infections

Viral RNA was extracted from plasma or serum samples derived from EDTA-clotted blood and from cerebrospinal fluid samples with the High Pure Viral Total Nucleic Acid Extraction Kit (Roche, Indianapolis, IN, USA). Nervous tissue or visceral organ samples were homogenized by using a mortar and pestle, and RNA was extracted by using the RNeasy Plus Mini Kit (QIAGEN, Valencia, CA, USA).

The first round and nested PCR primers that were based on a highly conserved region of the nsP4 gene were prepared as described previously (*1*). First-round PCR was performed by using the Titan one tube reverse transcription (RT)-PCR system (Roche). Each reaction contained 10 µL of RNA, 10 µL 10× reaction buffer, 1 µL of dNTP mix (10 mmol/L), 2 µL of 10 pmol of each primer (Alpha1+; Alpha1–), 2.5 µL Dithiothreitol solution (100 mmol/L), 0.25 µL RNase inhibitor (40 U/µL), and 1 µL of the Titan enzyme mix. The final volume was made up to 50 µL with distilled water. Cycling conditions were 50°C for 30 min, 94°C for 2 min, (94°C for 10 s, 52°C for 30 s, 68°C for 1 min) × 35 cycles, and 68°C for 7 min.

By using Primer 3 (*2*), two probes were designed for rapid differentiation of Sindbis virus (SINV, a New World virus) and Middelburg virus (MIDV, an Old World virus) as follows: SINV: 5′ ATGACGAGTATTGGGAGGAGTTTG 3′-FAM; MIDV: 5′ GCTTTAAGAAGTACGCATGCAACA 3′ –VIC. The probe position for MIDV is 6,132 by using MIDV EF536323 as reference and 6,478 for SINV (AF103734).

Differentiation of MIDV and SINV was performed on first-round RT-PCR products by real-time PCR by using the Lightcycler TaqMan Master kit (Roche): 2 μL of RT-PCR product was used with 1 μL of 20 pmol of each primer (Alpha2+; Alpha2–), 0.2 μL of 10 pmol probe each for SINV and MIDV, 4 μL of FastStart enzyme mixture to a final volume of 20 μL. The following program was followed on a Lightcycler 2 machine (Roche): preincubation at 95°C for 10 min (95°C for 10 s, 52°C for 1 min, 72°C for 1 s) for 45 cycles. Qualitative analysis that used different channels could distinguish between SINV and MIDV.

2. Alphavirus E-Protein Amplification

SINV E-Protein RT-PCR

Titan one tube RT-PCR system (Roche) was used for first-round PCR by using primer SIN8136EF (5′ TCGTCAGCATACGACATGGAG 3′) and A2 (*3*). PCR amplification began at 94°C for 2 min and 40 cycles of 94°C for 10 s, 52°C for 30 s and 72°C for 1 min with a final elongation step of 72°C for 7 min.

Expand High Fidelity^{PLUS} PCR system (Roche) was used for the semi-nested reaction. A total of 5μL of first-round product was used with primers SIN8136EF and SIN8787ER (5′GTATCCAAACTGGGCGGAAGT 3′). The following cycling program was used: 94°C for 2 min and 10 cycles of 94°C for 30 s, 52°C for 30 s, and 72°C for 1 min; 25 cycles of 94°C for 30 s, 52°C for 30 s, and 72°C for 1 min + 10 s/cycle with a final elongation step of 72°C for 7 min.

MIDV E-Protein RT-PCR

Titan one tube RT-PCR system (Roche) was used for first-round PCR with primer MID10475E+ (5′ GGTGCACGTTCCATATACCC 3′) and MID11045E- (5′ TCCCAATAGCAATCACCACA 3′). Cycling began at 94°C for 2 min and 40 cycles of 94°C for 30 s, 48°C for 45 s, and 72°C for 1 min with a final extension of 72°C for 7 min.

Nested PCR was performed with Expand High Fidelity^{PLUS} PCR system (Roche) by using 2μL of first round template, 5μL Q-solution (QIAGEN, Valencia, CA, USA), and primers MID10543EN+ (5′ TGAACCACAAGGCTCCTTTC 3′) and MID10911EN- (5′ CACTTTGCTGTGCAAGTGGT 3′). Cycling commenced at 94°C for 2 min and 40 cycles of 94°C for 30 s, 50°C for 45 s and 72°C for 1 min, with a final elongation step of 72°C for 7 min.

References

1. Sánchez-Seco MP, Rosario D, Quiroz E, Guzmán G, Tenorio A. A generic nested-RT-PCR followed by sequencing for detection and identification of members of the alphavirus genus. J Virol Methods. 2001;95:153–61. http://dx.doi.org/10.1016/S0166-0934(01)00306-8

2. Rozen S, Skaletsky H. Primer3 on the WWW for general users and for biologist programmers. Methods Mol Biol. 2000;132:365–86.

3. Lundström JO, Pfeffer M. Phylogeographic structure and evolutionary history of Sindbis virus. Vector Borne Zoonotic Dis. 2010;10:889–907. http://dx.doi.org/10.1089/vbz.2009.0069

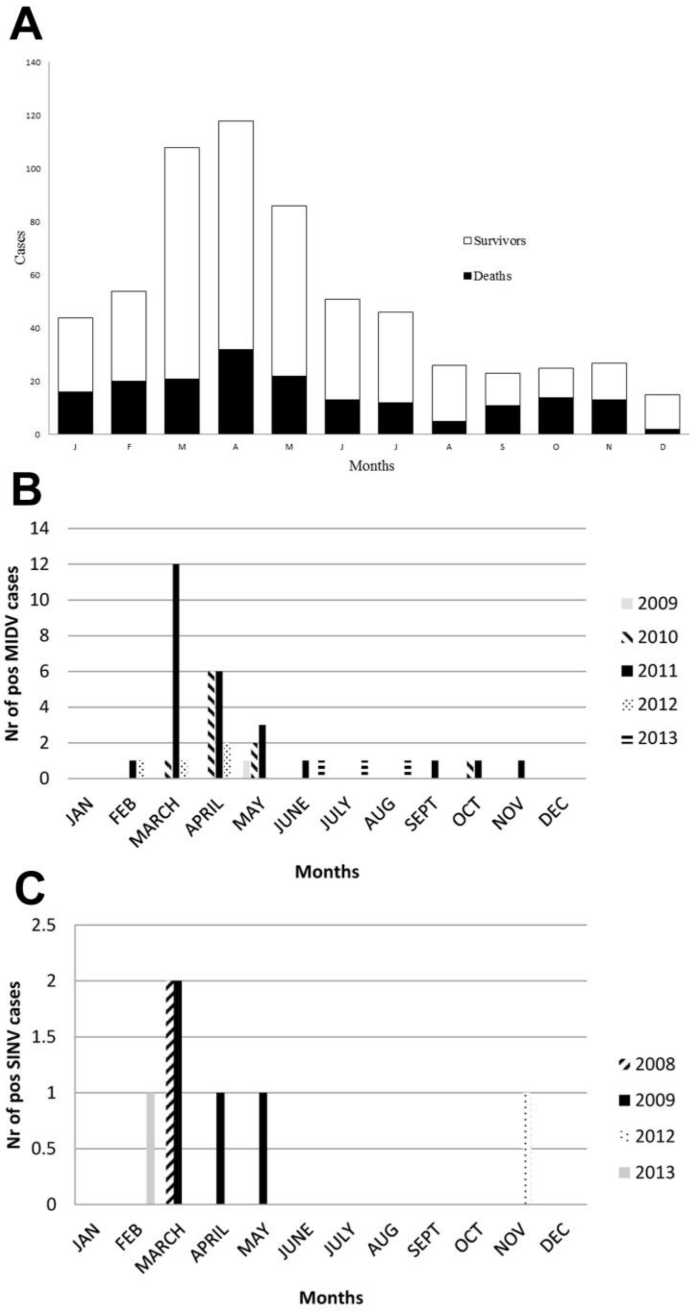

Technical Appendix Figure 1. Seasonal distribution of febrile and neurologic alphavirus–positive cases in South Africa. A) Bar chart showing monthly distribution of 623 cases of febrile and neurologic disease investigated in horses in South Africa during 2008–2013. Chart shows proportion of cases resulting in fatalities. B) Seasonal distribution of Middelburg virus (MIDV) cases investigated in horses in South Africa during 2008–2013. C) Seasonal distribution of Sindbis virus (SINV) cases investigated in horses and identified by reverse transcription PCR in South Africa during 2008–2013.

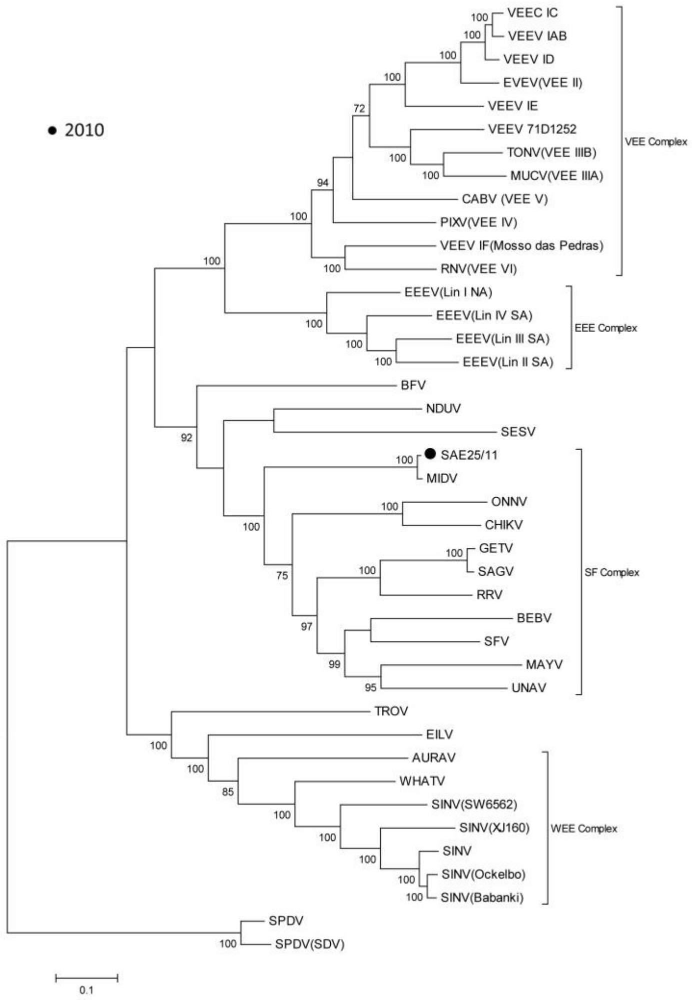

Technical Appendix Figure 2. A maximum-likelihood tree of the complete genome of Middelburg virus (MIDV SAE25/2011; KF680222) isolated in 2010 from a horse with neurologic disease, relative to strain MID-857 (identified as MIDV in the tree) isolated in 1993 from a horse that died with signs similar to those of African horse sickness virus. The tree was constructed with the Tamura-Nei substitution model by using the Mega 5 program (http://www.megasoftware.net/). Scale bar indicates 0.1 nt substitutions. Estimates were constructed on the basis of bootstrap resampling performed with 1,000 replicates. Complexes are identified as follows: EEE, Eastern equine encephalitis; SF, Semliki Forest; VEE, Venezuelan equine encephalitis; WEE, Western equine encephalitis. Viruses are identified as follows: BEBV, Bebaru virus; BFV, Barmah Forest virus; CHIKV, Chikungunya virus; EEEV, Eastern equine encephalitis virus; EVEV, Everglades virus; GETV, Getah virus; MAYV, Mayaro virus; MIDV, Middelburg virus; NDUV, Ndumu virus; ONNV, O'nyong nyong virus; RRV, Ross River virus; SAE, South Africa equine virus; SAGV, Sagiyama virus; SESV, Southern elephant seal virus; SFV, Semliki Forest virus; SINV, Sindbis virus; SPDV, Salmon pancreatic disease virus; TONV, Tonate virus; UNAV, Una virus; VEEV, Venezuelan equine encephalitis virus.

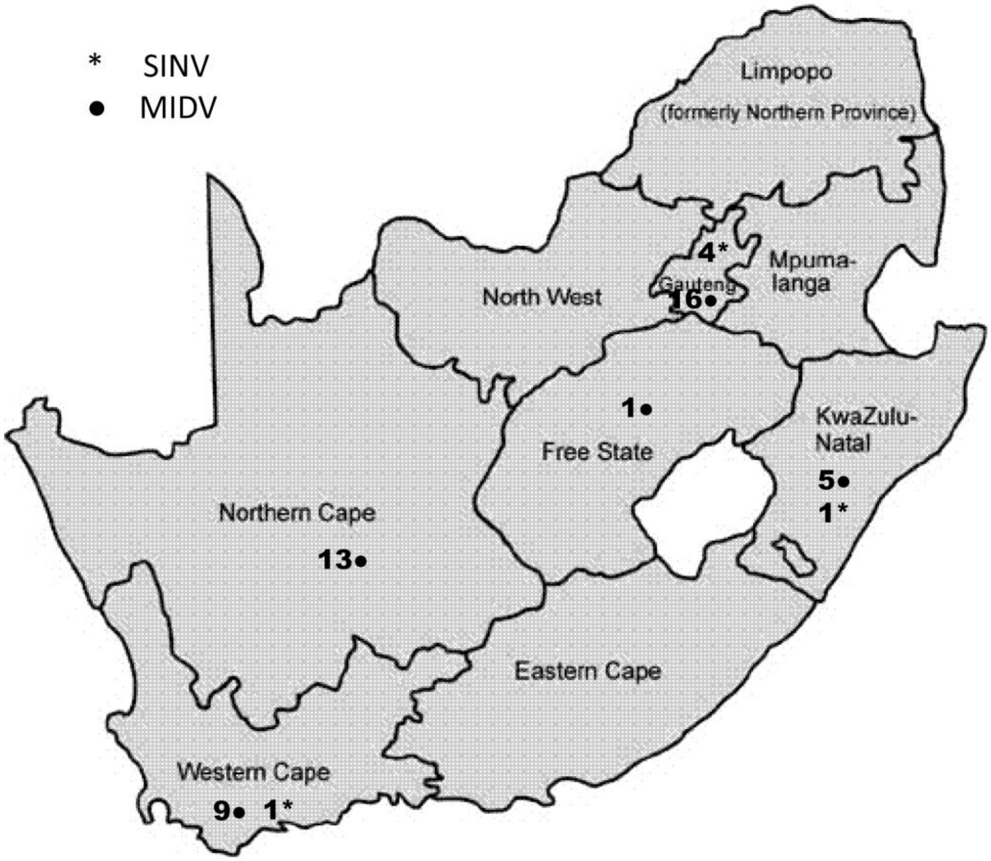

Technical Appendix Figure 3. Geographic distribution of alphavirus cases investigated in horses in South Africa, 2008–2013. Map shows numbers of cases of Sindbis virus (SINV) and Middelburg virus (MIDV) for specific locations. White area inside map indicates the independent nation of Lesotho.
